# The role of gut microbiota in depression: an analysis of the gut-brain axis

**DOI:** 10.3389/fnbeh.2023.1185522

**Published:** 2023-06-02

**Authors:** Natasha Irum, Tayyeba Afzal, Muhammad Hamid Faraz, Zeeshan Aslam, Faisal Rasheed

**Affiliations:** ^1^Medical Unit 02, Nishtar Medical University, Multan, Pakistan; ^2^Services Institute of Medical Sciences, Lahore, Pakistan; ^3^Central Park Medical College, Lahore, Pakistan; ^4^Nishtar Institute of Dentistry, Nishtar Medical University, Multan, Pakistan

**Keywords:** gut microbiota, depression, serotonin, gut-brain connection, gut-brain axis, gut normal flora

## Abstract

The gut-brain axis is a communication pathway that allows a two-way exchange of information between the microbiota of the gastrointestinal tract and the nervous system of humans. The vagus nerve, which is responsible for facilitating communication, provides support for this axis. The gut-brain axis is currently the subject of research, but studies into the diversity and stratification of the gut microbiota are just getting started. Researchers have discovered several positive trends by analyzing numerous studies examining the gut microbiota’s impact on the effectiveness of SSRIs. It is common knowledge that a specific group of measurable, microbial markers has been recognized as being present in the feces of individuals suffering from depression. Specific bacterial species are a common denominator among therapeutic bacteria used to treat depression. It can also play a role in determining the severity of disease progression. Evidence that selective serotonin reuptake inhibitors (SSRIs) rely on the vagus nerve to exert their therapeutic effects has provided further support for the importance of the vagus nerve in the gut-brain axis, which is necessary for beneficial changes in the gut microbiota. This review will analyze the research linking gut microbiota to depression.

## Introduction

Depression affects 21 percent of the world’s population (almost 280 million people) (Zhao et al., 2018; Chang et al., 2022), but for a number of different reasons, the condition is frequently misdiagnosed and left untreated. Persistent low mood characterizes depression, a prevalent psychiatric disorder that is hampered by social stigma, a shortage of effective treatments, and insufficient mental health resources. Depression is characterized by a persistently low mood lasting for at least 2 weeks (Zhao et al., 2018). Currently, selective serotonin reuptake inhibitors are the treatment of choice for major depressive disorders (MDD) (McGovern et al., 2019). Pleiotropic is the best way to describe the effect that serotonin (5-hydroxytryptamine, or 5-HT) has on diseases affecting the digestive tract, the neurological and psychiatric systems, the immune system, and the liver (Sherman et al., 2015; Liang et al., 2018; Stasi et al., 2019b). In the gut lumen, 5-HT serves multiple purposes, including the regulation of peristaltic reflexes, which are important to the digestive process. One of these functions is that 5-HT regulates the release of serotonin. The highest levels of 5-HT are located in the intestinal epithelium’s enterochromaffin (EC) cells. Mechanical stimulation of EC cells causes the release of 5-HT into the lumen, thereby enabling peristaltic reflexes. This response can be triggered by various stimuli, including nutrients (for example, glucose and short-chain fatty acids), acids, bases, and even mechanical stimulation (Hata et al., 2017).

The 400 to 1,000 species of bacteria that call the human gastrointestinal tract home are also known to have an effect on the functioning of the central nervous system. An increasing amount of attention is being paid to the mutually beneficial relationship that exists between the gut microbiota and their host (Sherman et al., 2015). People’s understanding of the concept of a connection between the gut and the brain via an axis has increased over the past few years. Advancements in the treatment of depression will result from research into the positive effects of gut microbiota on behavior and brain function (Cryan et al., 2019; McVey Neufeld et al., 2019; Liu et al., 2020). Oral therapy with *Lactobacillus rhamnosus* JB-1 has been shown to be effective in treating anxiolytic and antidepressant-like behaviors in clinical practice (McVey Neufeld et al., 2019). The gut-brain axis mediates this effect. However, some people think that the way SSRIs affect the gut rather than the drug itself is what actually causes the positive effects. Preventing serotonin from being taken up by neurons in the brain’s presynaptic region is central to how SSRIs work (McVey Neufeld et al., 2019).

By elucidating the influence of the gut microbiota and the interaction between antidepressants and the gut-brain axis, it may be possible to improve the availability of treatment options and preventive measures for individuals with major depressive disorder (MDD). Recent advances in the treatment of MDD have placed gut microbiota in the limelight, emphasizing its significance as an area of investigation (Siopi et al., 2020). By examining the effect of changes in gut microbiota on the efficacy of selective serotonin reuptake inhibitors (SSRIs) on the gut-brain axis, we can gain important insights into the treatment of depression and MDD.

It is important to note that the primary objective of research in this area is to identify additional effective treatments and preventive measures for major depressive disorder (MDD) through understanding the gut-brain axis and the role of gut microbiota in the development of depression and its treatment. This is important to note because it further emphasizes the review’s objectives. The high prevalence of depression, combined with social stigma, an absence of effective treatments, and insufficient resources for mental health, demonstrates the urgent need for more effective treatments for this disorder.

It has been shown that serotonin (5-HT), which is regulated by the microbiota in the gut, plays an important role in both the development of depression and its treatment.

Additional research needs to be conducted in this area to advance our knowledge of the gut-brain axis, the role of gut microbiota in depression, and the efficacy of SSRIs. This review may pave the way for developing innovative antidepressant treatments concentrating on gut microbiota. In addition, it highlights the significance of having a healthy microbiome in the gut for mental health and overall well-being.

## A comprehensive review

### Depressive gut microbiota

Gut microbiota has been separated into healthy microbiota in a number of ways that have helped scientists tell the difference between pathology and physiology—the distinction between healthy and disease-causing vegetation allowed for the development of the concept of stool signatures. Changes in the gut microbiota have been shown in several studies to be the cause of differences in stool composition between healthy subjects and depressed patients. In an experiment that Liu et al. (2020) carried out, the fecal microbiota of both depressed patients and healthy individuals were transplanted into rats that had been germ-free. They found that when rats were given the fecal microbiota of depressed patients, the rats developed depressive symptoms (Liu et al., 2020). The manipulation of gut microbiota can influence brain chemistry, as evidenced by the successful induction of depression through fecal transplantation. This manipulative concept is investigated by Fond et al. (2020) who suggest that fecal microbiota transplantation may be a viable treatment option for schizophrenia and major depressive disorder. The researchers contend that treatments focused on the microbiota are a promising approach to treat mental health conditions like schizophrenia and depression and that the use of capsules has helped reduce the risks associated with the procedure of fecal microbiota transplantation (Fond et al., 2020). Alterations in stool composition are another effect that may occur when taking antidepressants. Stool samples were collected prior to treatment in Bharwani et al. (2020) study. Three and 6 months after treatment with antidepressants, the participants provided blood and urine samples to show whether or not the treatment had been effective. When analyzing the response to treatment, the changes in stool revealed substantial differences within the microbial community. Despite the inclusion of participants with comparable initial depression severity scores, those who did not experience remission had a lower microbial diversity prior to the initiation of treatment. In addition, the abundance levels of 22 distinct microbial groups varied between the two response groups. Even after 6 months of treatment, the disparities observed at baseline between individuals who achieved remission and those who did not persisted: the remitters displayed consistently higher levels of microbial diversity (Bharwani et al., 2020). These alterations in stool composition brought on by antidepressant treatment give credibility to the phenotyping of depressive gut microbiota. Depressed people often experience these shifts as a sign that their gut microbiota is being restored.

When comparing the gut microbiota of unmedicated people with major depressive disorder (MDD), people with bipolar disorder, and healthy controls, Zheng et al. (2020) conclude that there are differences between the groups. This research had two groups of controls: one was similar to the study participants regarding the aforementioned demographic factors, while the other was not. The gut microbiota of MDD patients differed from that of controls with the same age, gender, and body mass index. There were more Enterobacteriaceae and Alistipes in MDD patients’ guts and fewer Faecalibacterium, Coprococcus, and Dialister. Individuals with MDD had their gut microbiota studied. In addition to this, they found that only Pseudomonadaceae levels were higher in subjects diagnosed with bipolar disorder when compared to healthy controls. A signature of 26 operational taxonomic units (OTUs) was found to be needed to tell major depressive disorder (MDD) apart from bipolar disorder and healthy controls. Four of the 26 microbial OTUs were significantly linked to the Hamilton Depression Rating Scale (HAMD) in people with major depressive disorder or bipolar disorder (Zheng et al., 2020). Most of these were from the family Lachnospiraceae. The optimal conditions for a healthy microbiota community in the gut can be better understood by conducting research that also considers the effects of pharmacotherapy and diet. Clinical interviews and symptomatology play a large part in contemporary methods for diagnosing mental health conditions. Further research into the microbial signatures in the gut that are unique to mental conditions such as major depressive disorder (MDD) may result in the development of a diagnostic test that is both quantifiable and has a higher cumulative diagnostic standard.

### Vagus nerve functions in the gut-brain axis

**FIGURE 1:**
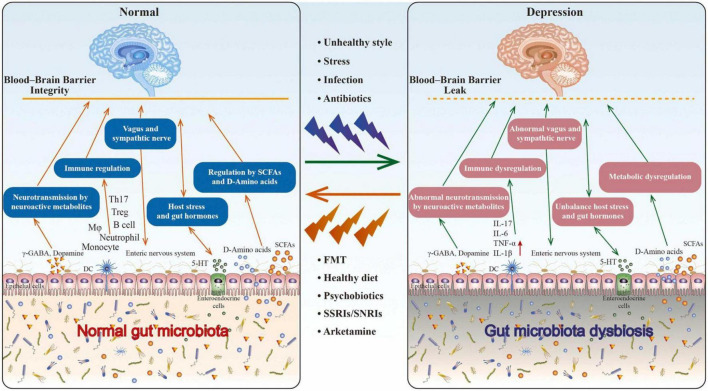
The role of gut microbiota in depression. Through the brain-gut-microbiota axis, research indicates that an unhealthy lifestyle caused by factors such as stress and infection can lead to dysbiosis in the gut microbiota, which can contribute to depression. Nonetheless, diverse methods, such as fecal microbiota transplantation, diet, psychobiotics, and antidepressants, can help restore the gut microbiota’s equilibrium and alleviate depressive symptoms. Promoting the growth of beneficial bacteria in the gut, producing neurotransmitters that positively impact brain function, and altering the levels of specific neurotransmitters in the brain are the mechanisms by which these approaches function. Restoring gut microbiota balance is a promising method for enhancing mental health. Taken from article (Chang et al., 2022).

The vagus nerve is the pathway through which the brain communicates with the digestive system. Because of this, the vagus nerve is the axis that is responsible for the gut-brain axis as also shown in Figure 1 (Breit et al., 2018). Notably, SSRIs not only modulate serotonin levels in the stomach but also target the pivotal role played by the vagus nerve in mood control. McVey Neufeld et al. (2019) researched on the effectiveness of SSRIs in mice with and without vagotomies. Recordings made from the mesenteric nerve revealed that orally administered SSRIs raised the level of vagal activity in the control group. The subdiaphragmatic vagotomized mice performed poorly in the tail suspension test, resulting in poor overall results, indicating that the antidepressant effects of SSRI treatment were not present in them (McVey Neufeld et al., 2019). These results demonstrate that the vagus nerve is necessary for SSRIs to have their therapeutic effect. This is the case even though these medications are used. Bravo et al. (2011) also demonstrated that subdiaphragmatic vagotomy inhibited the anxiolytic effects of *L. rhamnosus* in mice. Because Bravo et al. (2011) performed vagotomies on mice in a subdiaphragmatic location, they were able to isolate the vagus’s function from the gut-brain axis. Effective management requires protecting the vagus nerve due to its significance in the gut-brain axis and because selective serotonin reuptake inhibitors (SSRIs) and anxiolytic bacteria, bacteria that have been identified as having potential anxiety-relieving or calming effects, rely on it.

The vagus nerve’s role in the gut-brain axis has been well established, and now emerging evidence suggests that BDNF levels in the hippocampus also play a crucial role in this intricate communication system. According to Bercik et al. (2011), cutting the vagus nerve significantly affects BDNF (brain-derived neurotrophic factor) levels in the hippocampus. Recent evidence indicates that the microbiota plays a role in this process. BDNF is an essential protein involved in the growth and maintenance of neurons, specifically in the hippocampus. When the vagus nerve is severed, communication between the gut and brain is disrupted, resulting in altered BDNF levels. Emerging evidence indicates, however, that the gut microbiota may also regulate BDNF levels in the hippocampus. Further study is required to comprehend the intricate interplay between the vagus nerve, microbiota, and BDNF levels in the hippocampus and how these interactions may affect brain function and cognition.

### *Lactobacillus* is good for the digestive system

It has been determined that a change in the bacteria in the gut is a helpful step toward alleviating depression. These parameters are related to metabolic pathways or their suppressive effects on inflammatory markers. It has been demonstrated in a great number of studies that the *Lactobacillus* in the microbiota of the gut has a beneficial impact on patients suffering from MDD (Heym et al., 2019). In a study that Capuco et al. (2020) did, they looked at how effective a placebo, an SSRI, and a probiotic with *Lactobacillus plantarum* 299v were at treating the symptoms of depression. The study was designed as a double-blind, placebo-controlled experiment. In the treatment group, neurotoxic and neurodegenerative amino acid kynurenine levels were significantly reduced (Capuco et al., 2020).

In comparison to the initial state, results from tests of attention, perceptiveness, and verbal learning all showed signs of improvement in cognitive function. Tryptophan can participate in the synthesis of serotonin or kynurenine, depending on which metabolic pathway it is directed toward. A mechanism very similar to that of SSRIs is utilized by probiotics, in which tryptophan is redirected toward the production of serotonin. This results in an increase in serotonin level and a reduction in depressive symptoms (Capuco et al., 2020). In addition, Han and Kim (2019) demonstrate that administering *Bifidobacterium longum* NK46 and *Lactobacillus mucosae* NK41 probiotic strains can inhibit the activation of tumor necrosis factor-alpha (TNF) and nuclear factor kappa B (NF-B). TNF and NF-B are both components involved in the pathogenicity of depression in depression- and anxiety-induced mice. Both of these probiotic strains were administered to anxiety- and depression-induced mice (Han and Kim, 2019).

In addition, the combination of NK41 and NK46 was shown to significantly reduce the anxious and depressive behaviors that are brought on by immobilization stress (Han and Kim, 2019). Researchers, Yong et al. (2020), found that chronically stressed mice with *L. rhamnosus* in their guts displayed less depressive-like behavior than mice without the probiotic. To evaluate the effects of probiotic therapy, a comprehensive battery of behavioral tests was administered in this study (Bravo et al., 2011). In particular, the stress-induced hyperthermia (SIH) and elevated plus maze (EPM) tests were used, which are commonly used to assess the functional consequences of alterations in GABA neurotransmission. Chronic administration of *L. rhamnosus* (JB-1) resulted in a non-significant reduction in stress-induced hyperthermia, indicating a potential stress-reducing effect. In the EPM test, animals treated with *L. rhamnosus* (JB-1) demonstrated a more significant number of entries than the control group, indicating the probiotic’s anxiolytic properties. These findings suggest that non-pathogenic bacteria, such as *L. rhamnosus* (JB-1), can modulate the GABAergic system, which may have therapeutic applications in treating depression and anxiety.

Moreover, it is noteworthy that these effects were observed in healthy animals. In contrast, most previous studies investigating potential probiotics’ effects on the microbiome-gut-brain axis relied on infected, germ-free, or antibiotic-treated animals. Therefore, these findings make it more challenging to comprehend the therapeutic potential of bacteria in modulating brain function and behavior. Changes in GABA receptor subunit-related transcripts provide mechanistic insights into the effect of *L. rhamnosus* (JB-1) on anxiety-like behavior. The involvement of other neurotransmitter and neuropeptide systems, such as 5-hydroxytryptamine, norepinephrine, glutamate, and corticotropin-releasing factor, cannot be ignored and should be investigated in future research. In addition, research should determine the duration of these alterations and whether *L. rhamnosus* (JB-1) modulates other systems. The vagus nerve plays a key role in mediating the behavioral and molecular changes induced by *L. rhamnosus* (JB-1), establishing a clear communication pathway between bacteria, the gut, and the brain that influences responses to various stress-related situations.

These studies provide evidence that a particular strain of *Lactobacillus* is effective in treating major depressive disorder (MDD) via anti-inflammatory mechanisms and by stimulating serotonin production. More research on the synergistic effects of SSRIs and the possible synergistic effects of giving *Lactobacillus* through probiotics can help improve the treatment plan for major depressive disorder (MDD).

## The role of the gut-brain axis in conditions other than depression

The gut-brain axis is involved not only in depression but also in reactions to psychiatric drugs, vertically during pregnancy, irritable bowel syndrome (IBS), autism, and many other conditions. Because of its responsiveness to probiotics, irritable bowel syndrome (IBS) is often cited as the prototypical disorder of the gut-brain axis (Dinan and Cryan, 2017). A number of studies have shown that SSRIs are helpful for patients who suffer from IBS. Irritable bowel syndrome (IBS) patients who took the SSRI fluoxetine had significantly improved gastrointestinal and psychiatric symptoms, according to the findings of a study conducted by Stasi et al. (2019a) that was conducted over the course of several years. The fact that psychiatric and gastrointestinal symptoms improved simultaneously demonstrates that mental screening has the potential to improve care for all patients suffering from IBS. Up to 10 percent of pregnant women take selective serotonin reuptake inhibitors (SSRIs) for depression, which can have an effect on the development of the brain in the fetus and the gut-brain axis (Ramsteijn et al., 2020; Salisbury et al., 2020). Davey et al. (2013) found that olanzapine could be used to treat metabolic dysfunction caused by antipsychotics. This is because olanzapine has a similar effect on the gut-brain axis as selective serotonin reuptake inhibitors. Sherman et al. (2015) think it would be good to learn more about the gut-brain axis in newborns. According to the findings of their study, the treatment of premature infants with necrotizing enterocolitis with probiotic bacteria resulted in significant improvements (Sherman et al., 2015). Researchers have demonstrated that probiotics prevent brain damage by inhibiting the transport of harmful biomolecules, which is one of the benefits of taking them. Clarke et al. (2013) the study’s authors, also talk about how the gut-brain axis plays a part in the development of the immune and endocrine systems. They discover evidence that re-establishing a healthy digestive system in the elderly may not be beneficial (Clarke et al., 2013). Beginning in preterm infanthood and continuing well into adulthood, it is possible to observe the significance of the gut-brain axis. According to recent findings, modifying the gut-brain axis can be an effective treatment for various diseases, including Parkinson’s, multiple sclerosis, autism, and obesity (Cryan and Dinan, 2012; Santos et al., 2019; Srikantha and Mohajeri, 2019).

Santos et al. (2019) found that the way alpha-synuclein accumulates in the central and peripheral nervous systems, which is a sign of Parkinson’s disease, can be caused by toxins in the gut that are transmitted to the CNS through the vagus nerve. Srikantha and Mohajeri (2019) looked into the connection between ASD and gastrointestinal problems by studying the gut-brain axis. The findings suggest that children with ASD have a more limited range of microorganisms in their bodies compared to healthy controls. The microbiome of someone with autism spectrum disorder is characterized by a decrease in the ratio of Bacteroidetes to Firmicutes, in addition to other characteristics, in addition to the fact that there is a lack of diversity. In the same way that the microbiota of MDD has been given a “signature,” Srikantha and Mohajeri (2019) have found that the microbiota of people with ASD has its own unique traits. Again, there is evidence that the gut microbiota is important to the cause of mental health problems and ASD (Mangiola et al., 2016). Understanding the relationship between the gut-brain axis, microbiota, and probiotics, can pave the way for novel approaches to preventing and treating diseases across many medical specialties, which is good news for patients of all ages.

## Conclusion

A growing body of research is being conducted to elucidate the function of the gut microbiota with depression and the use of antibiotics. There have been a number of studies that point to the encouraging implications of optimizing depression treatment by customizing the gut microbiota. Studies have shown that there is a unique pattern of bacteria in the stool that can help confirm a clinical diagnosis of depression. Stool samples are useful for this purpose. Because this phenotype has been defined, researchers are now able to concentrate on finding ways to incorporate it into diagnostics. This is especially important for the numerous psychiatric disorders for which there are no quantifiable measures. In addition, for the treatment of MDD, we present potential treatments that involve fecal transplants and have only minimal adverse effects. It has been demonstrated that it is beneficial to combine the administration of SSRIs with probiotics that contain specific characteristic bacteria. The axis connecting the gut and the brain has made these discoveries possible. The significance of the gut-brain axis in allowing the transmission of these implications was determined by comparing mice with and without vagotomies. This allowed the researchers to determine the significance of the gut-brain axis. Evidence suggests that promoting healthy gut microbiota can improve the management of irritable bowel syndrome (IBS), as SSRIs have been shown to affect patients with IBS positively via the gut-brain axis. Establishing a stool signature for depression has been shown to contribute to the diagnostic process, which currently relies heavily on patient interviews and narrow differential diagnoses. This is something that is important because the diagnostic process for depression currently relies heavily on patient interviews. Researchers can create probiotics and give them to patients to restore a healthy gut microbiome once they have identified the stool signature of depression. Antidepressants and the gut microbiota are still being studied as potential treatment areas. With the help of this study, we can now see how viruses and fungi contribute to the digestive system’s ecosystem. More research into the effects of lactose-free diets, healthy diets, the role of fungi and viruses in the gut-brain axis, and the evidence-based application of stool signatures in the diagnosis of depression would be very welcome.

## Author contributions

NI, TA, and MF: manuscript writing. FR: proofread and editing. All authors contributed to the article and approved the submitted version.
